# The Southern Dental Association—1893

**Published:** 1893-12

**Authors:** 


### The Southern Dental Association.—1893.

Pursuant to call, the Southern Dental Association convened in Chicago, August 11, 1893.

Dr B. Holly Smith, Baltimore, President, in the chair.

The meeting was called to order at 11 a. m.

On motion, reading of the minutes of the last meeting was dispensed with.

The Executive Committee reported as follows:

That the association adjourn till the next annual meeting, all officers and committees holding over till that time; that no dues be required for the present year; that the books of the treasurer and secretary had been examined and found correct.

The Treasurer, Dr. Beach, directed attention to the by-law of the association requiring that each member pay three dollars a year dues. He did not think they could legally remit the dues for the year, but they could amend the rule requiring each member to pay his dues before he took any part in the meeting. He thought that it would be necessary for members to pay their dues during the year if they were to continue in good standing.

Dr. McKellops thought that in these extraordinary times of financial crisis the association had a perfect right to act in the premises and to act at once.

Dr. S. W. Foster, Secretary, said that by unanimous consent the by-laws could be suspended.

This was then put in the form of a motion and carried unanimously, and all dues for 1893 remitted.

The Executive Committee reported that charges were brought against Dr. E. B. Marshall, of Rome, Ga., of unprofessional conduct in having advertised, and the secretary read certain advertisements attached to the charges.

On motion, Drs. Gordon White, N. A. Williams and S. W. Foster were appointed a committee to correspond with Dr. Marshall in regard to this matter, with instructions to report at the next annual meeting.

The reports of all other committees were deferred until the next annual meeting, all committees holding over.

The secretary presented bills for stationery, etc., which on motion, were ordered paid.

Dr. Gordon White moved that the secretary and treasurer correct the list of members and prepare a new book. Carried.

On motion of Dr. McKellops the officers were allowed to hold over till the next annual meeting.

Dr. Marshall moved that the selection of the place of the next annual meeting be left to the Executive Committee and that the committee be instructed to report within six months. Carried.

On motion, the association adjourned till the next annual meeting.

